# Supplementary material for: Rewiring glucose metabolism improves 5-FU efficacy in p53-deficient/KRASG12D glycolytic colorectal tumors
Source: Commun Biol. 2022 Oct 31;5:1159. doi: 10.1038/s42003-022-04055-8 (PMC9622833; doi:10.1038/s42003-022-04055-8)
Supplement: Supplementary file 2 — Supplementary Information [file 42003_2022_4055_MOESM2_ESM.pdf]

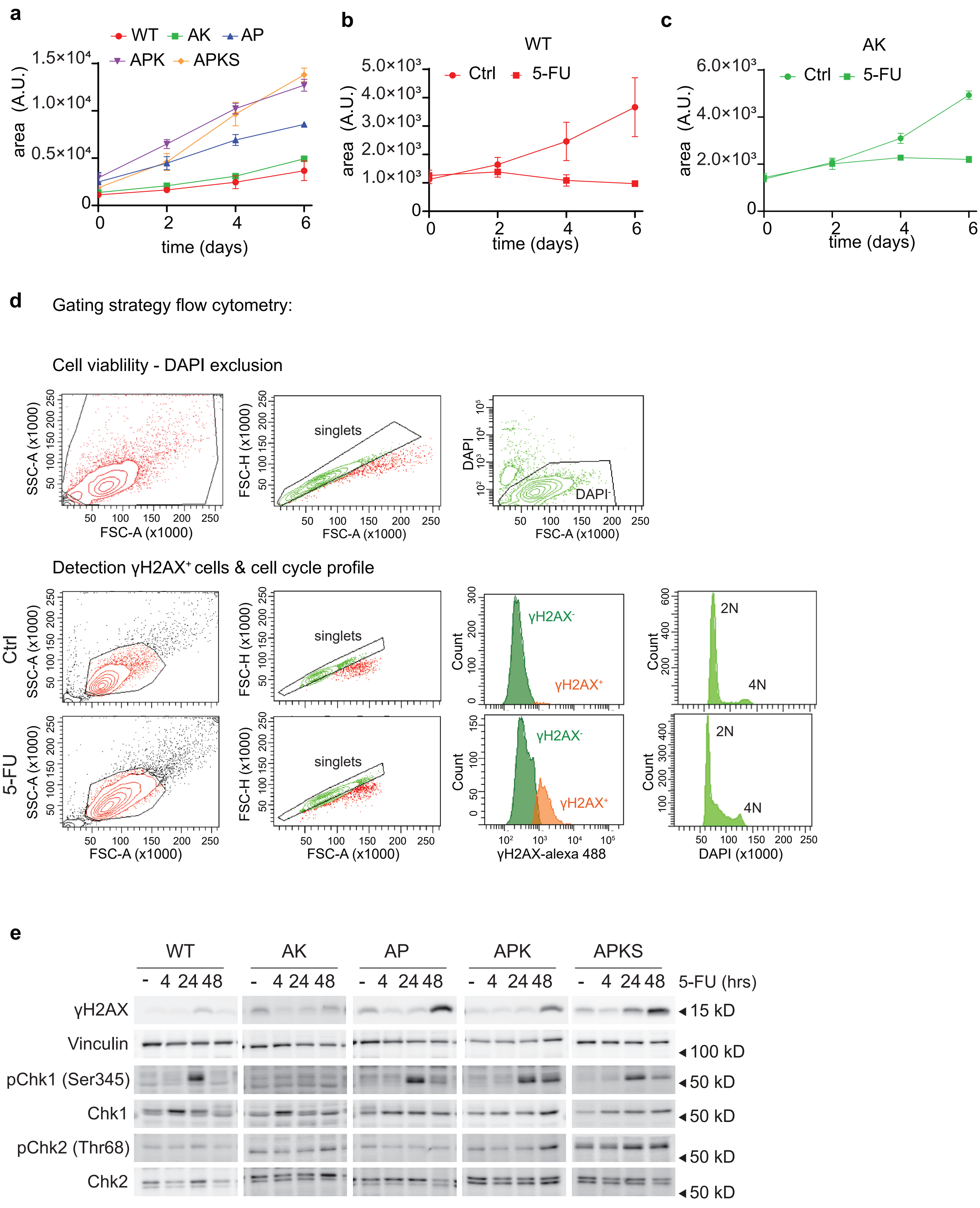
a
WT AK AP
APK APKS
area (A.U.)
time (days)
b
WT
Ctrl 5-FU
area (A.U.)
time (days)
c
AK
Ctrl 5-FU
area (A.U.)
time (days)
d
Gating strategy flow cytometry:
Cell viablility - DAPI exclusion
SSC-A (x1000)
FSC-A (x1000)
FSC-H (x1000)
singlets
DAPI
DAPI-
Detection γH2AX+ cells & cell cycle profile
Ctrl
5-FU
γH2AX-
γH2AX+
Count
γH2AX-alexa 488
2N
4N
DAPI (x1000)
e
WT AK AP APK APKS
- 4 24 48
5-FU (hrs)
γH2AX
Vinculin
pChk1 (Ser345)
Chk1
pChk2 (Thr68)
Chk2
15 kD
100 kD
50 kD

Supplementary Fig. 1. Growth curves, flow cytometry gating strategy & 5-FU time course. a) Area of WT and CRC organoids during 6 days of culturing in differentiation medium (mean ± SEM, >150 organoids from two independent experiments). **b** and **c)** Area of 5-FU-treated WT (B) and AK (C) organoids during 6 days of culturing in differentiation medium (mean ± SEM, >150 organoids from two independent experiments, data of untreated controls is the same as in A). **d)** Flow cytometry gating strategies for cell viability analysis by DAPI exclusion, detection of $\gamma H2AX^+$ cells and cell cycle profile analysis. **e)** Western blot detection of (p)Chk1, (p)Chk2, γH2AX and vinculin of lysates from WT and CRC organoids treated with 5-FU for 4, 24 or 48 hours (representative for n = 3, Chk2: Santa Cruz, #SC-9064). WT, AP and APK were run together on the same gels. AK and APKS were run on separate gels.

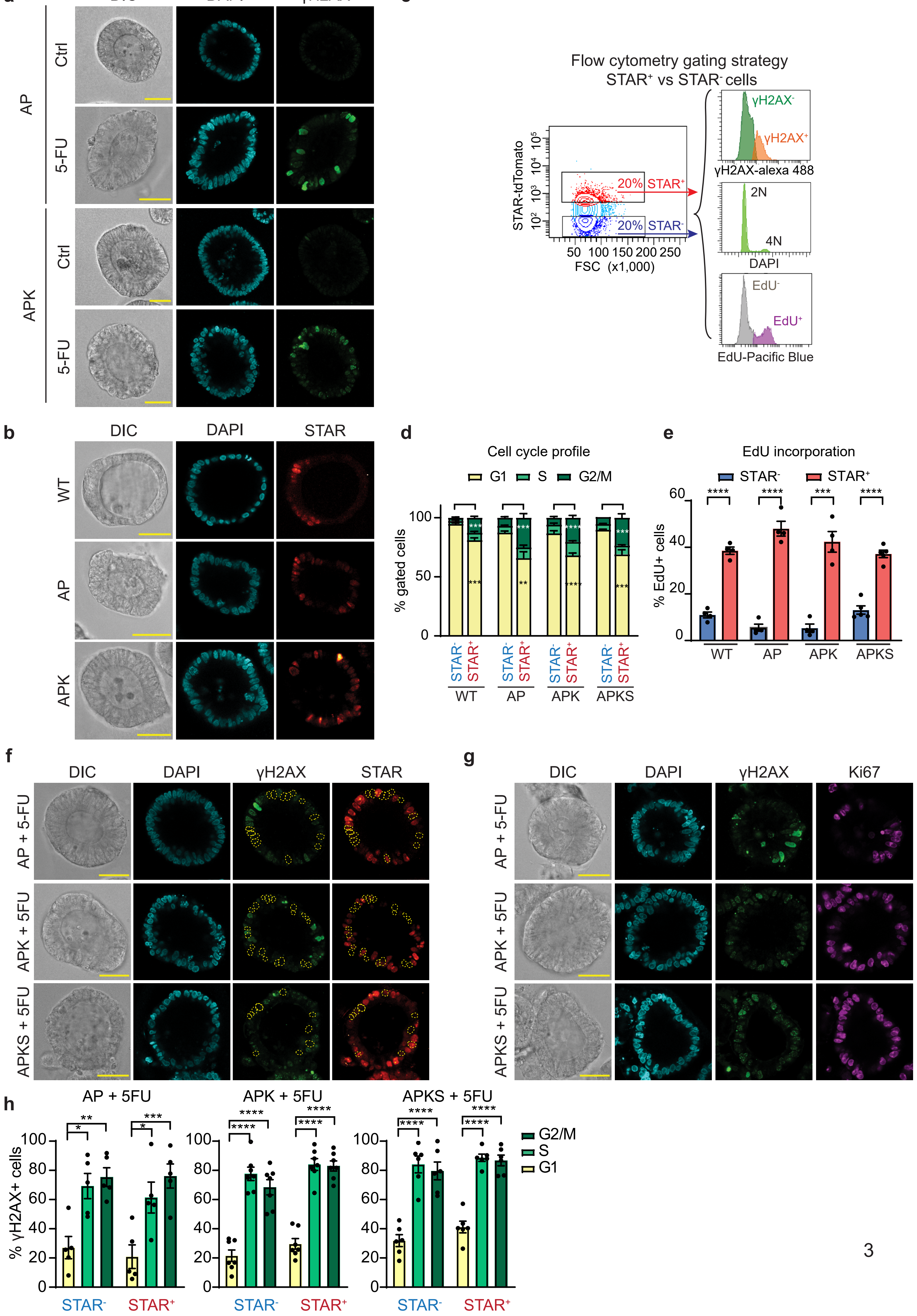
a
DIC
DAPI
γH2AX
AP
Ctrl
5-FU
APK
Ctrl
5-FU
b
DIC
DAPI
STAR
WT
AP
APK
c
Flow cytometry gating strategy
STAR⁺ vs STAR⁻ cells
STAR-tdTomato
FSC (x1,000)
20% STAR⁺
20% STAR⁻
γH2AX⁻
γH2AX⁺
γH2AX-alexa 488
2N
4N
DAPI
EdU⁻
EdU⁺
EdU-Pacific Blue
d
Cell cycle profile
G1
S
G2/M
% gated cells
STAR⁻
STAR⁺
WT
AP
APK
APKS
e
EdU incorporation
STAR⁻
STAR⁺
% EdU+ cells
WT
AP
APK
APKS
f
DIC
DAPI
γH2AX
STAR
AP + 5-FU
APK + 5FU
APKS + 5FU
g
DIC
DAPI
γH2AX
Ki67
AP + 5-FU
APK + 5FU
APKS + 5FU
h
AP + 5FU
APK + 5FU
APKS + 5FU
% γH2AX+ cells
G2/M
S
G1
STAR⁻
STAR⁺

Supplementary Fig. 2. 5-FU induces DNA damage in proliferating cells. a) Representative images of AP and APK organoids treated with 5-FU for 48 hours and stained with anti-γH2AX and DAPI (scale bar = 50 μm). **b)** Representative images of WT, AP and APK organoids transduced with the stem cell reporter STAR and stained with DAPI (scale bar = 50 μm). **c)** Flow cytometry gating strategy for DNA damage, cell cycle profile and EdU incorporation analysis in $STAR^+$ vs $STAR^-$ populations. **d)** Cell cycle profile determined by flow cytometry in $STAR^+$ vs $STAR^-$ cells of WT and CRC organoids (mean ± SEM, n = 4-6, unpaired t-tests). **e)** EdU incorporation analysis by flow cytometry in $STAR^-$ vs STAR+ cells in WT and CRC organoids (mean ± SEM, n = 4-5, one-way ANOVA, Sidak's multiple comparisons test). **f)** Representative images of AP, APK and APKS organoids transduced with the stem cell reporter STAR treated with 5-FU for 48 hours and stained with anti-γH2AX and DAPI (scale bar = 50 μm, dashed circles indicate $γH2AX^-$ nuclei). **g)** Representative images of AP, APK and APKS organoids treated with 5-FU for 48 hours and stained with anti-KI67 and DAPI (scale bar = 50 μm). **h)** Detection of $γH2AX^+$ cells by flow cytometry G1, S and G2/M cells in the $STAR^-$ and $STAR^+$ population of CRC organoids treated with 5-FU for 48 hours. Percentages $γH2AX^+$ cells are normalized per specific cell type (mean ± SEM, AP: n = 5, APK: n = 7, APKS n = 6, one-way ANOVA, Sidak's multiple comparisons test).

* p < 0.05, ** p < 0.01, , ** p < 0.01, *** p < 0.001, **** p < 0.0001.

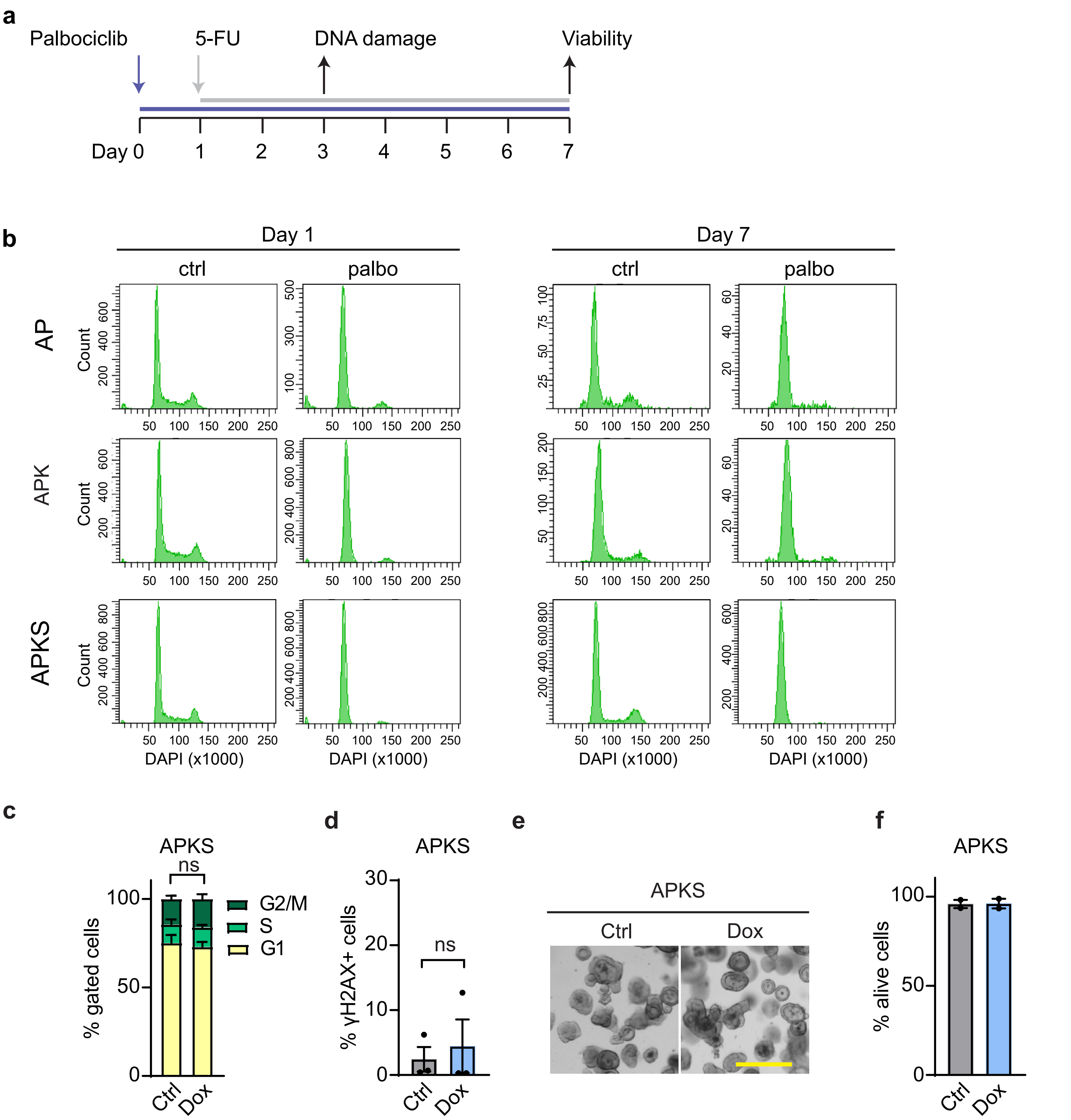

Supplementary Fig. 3. G1 arrest in AP, APK and APKS organoids by palbociclib treatment and doxycycline controls in APKS. a) Schematic overview of experimental set-up of experiments to determine DNA damage and viability upon palbociclib and 5-FU treatments. **b**) Flow cytometry cell cycle profiles of AP, APK and APKS organoids stained with DAPI and treated with palbocilib for 24 hours and 7 days. **c** and **d)**, Cell cycle profile and quantification of cells with DNA damage by flow cytometry of APKS organoids treated with doxycycline (200 ng/ml) for 24 hours and stained with DAPI (F) and anti-γH2AX (G) (F: mean ± SEM, n =2, G: mean ± SEM, n = 3, unpaired t-test). **e** and **f)** Brightfield images (**e**) and cell viability analysis (**f**) of APKS organoids treated with doxycycline (200 ng/ml) for 6 days (scale bar = 500 µm, mean ± SEM, n = 2).

Ns: non-significant

Supplementary Fig. 4

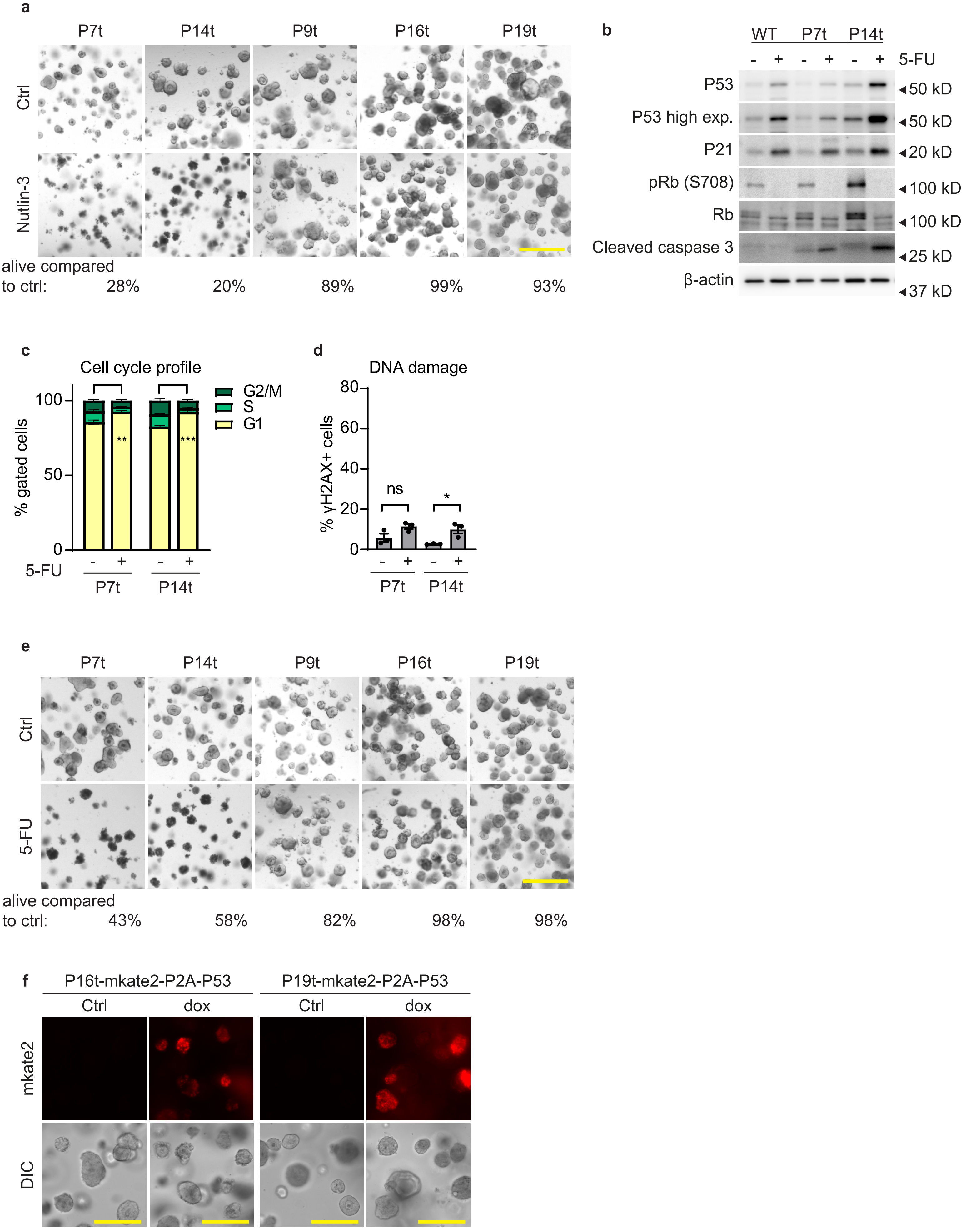

Supplementary Fig. 4. PDO characterization, 5FU response in in PDOs with functional P53 and P53-OE system in PDOs. a) Brightfield images of P7t, P14t (p53-functional), P9t, P16t and P19bt (p53 non-functional) organoids treated with Nutlin-3 (20 µM) for 72 hours. Relative cell viability was determined by DAPI exclusion analysis by flow cytometry (scale bar = 500 µm). **b)** Western blot detection of p53, p21, pRb (S708), Rb, cleaved caspase 3 and β-actin in WT, P7t and P14t organoids treated with 5-FU for 48 hours (representative for n = 3). WT, P7t and P14t were run together on the same gels. **c** and **d)** Cell cycle profile analysis (d) and Quantification of cells with DNA damage (d), of P7t and P14t organoids treated with 5-FU for 48 hours (c: mean ± SEM, n = 3, one-way ANOVA, Sidak's multiple comparisons test, d: mean ± SEM, n = 3-4, unpaired t-test. **e)** Brightfield images and cell viability analysis of P7t, P14t, P9t, P16t and P19bt organoids treated with 5-FU for 48 hours. Relative cell viability was determined by DAPI exclusion by flow cytometry (scale bar = 500 µm. **f)** Mkate2 and Brightfield images of doxycycline-inducible P16t- and P19bt-mkate2-P2A-P53OE organoids treated with doxycycline (200 ng/ml) for 48 hours (scale bar = 500 µm).

ns: non-significant, * $p < 0.05$, ** $p < 0.01$, *** $p < 0.001$

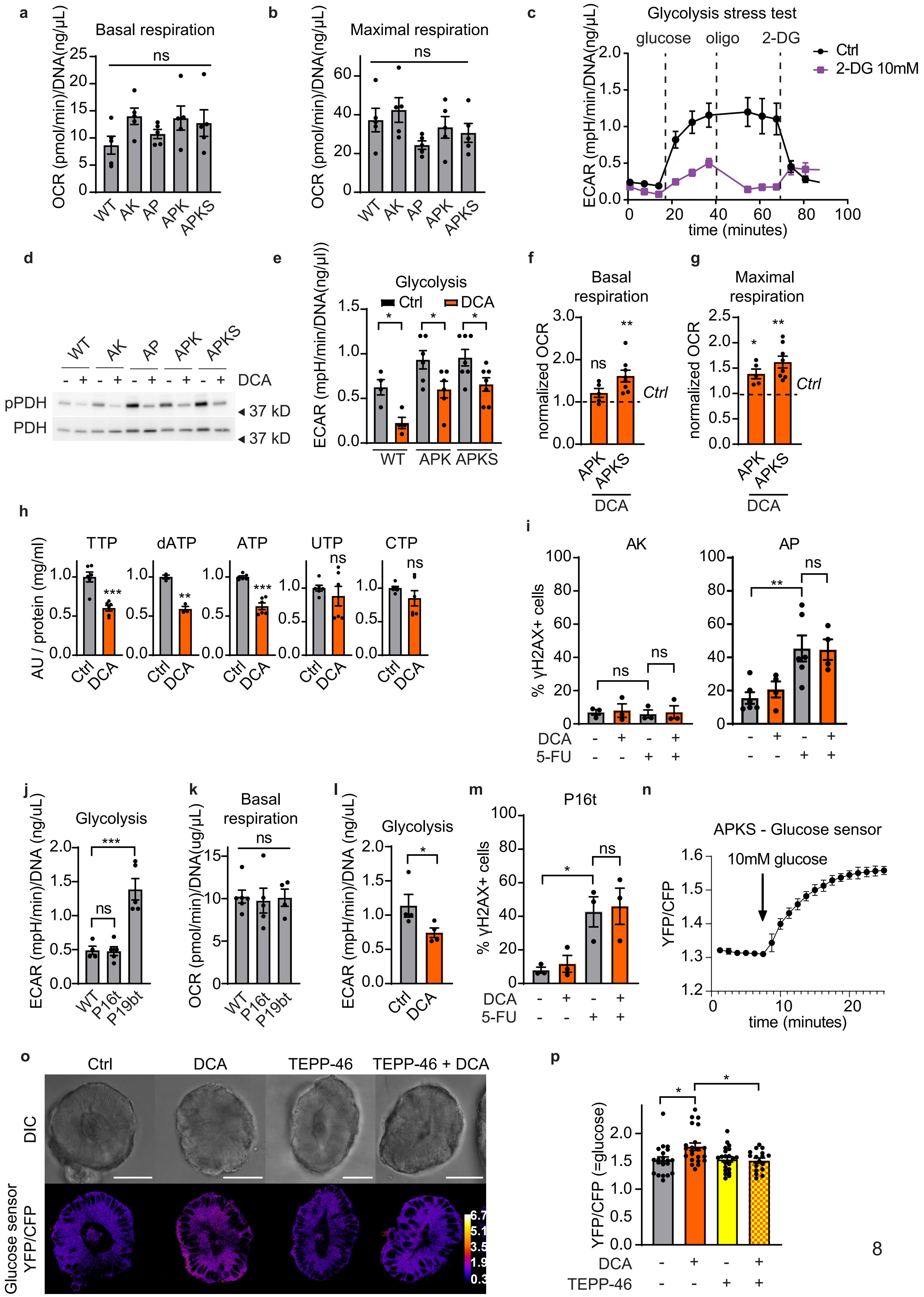
a
Basal respiration
OCR (pmol/min)/DNA(ng/µL)
ns
WT AK AP APK APKS
b
Maximal respiration
OCR (pmol/min)/DNA(ng/µL)
ns
WT AK AP APK APKS
c
Glycolysis stress test
glucose oligo 2-DG
Ctrl
2-DG 10mM
ECAR (mpH/min/DNA(ng/µL)
time (minutes)
d
WT AK AP APK APKS
DCA
pPDH
PDH
37 kD
37 kD
e
Glycolysis
Ctrl DCA
ECAR (mpH/min/DNA(ng/µl))
WT APK APKS
f
Basal respiration
normalized OCR
ns
Ctrl
APK APKS
DCA
g
Maximal respiration
normalized OCR
Ctrl
APK APKS
DCA
h
TTP dATP ATP UTP CTP
AU / protein (mg/ml)
ns
Ctrl DCA
i
AK AP
% γH2AX+ cells
ns
DCA
5-FU
j
Glycolysis
ECAR (mpH/min)/DNA (ng/uL)
ns
WT P16t P19bt
k
Basal respiration
ns
OCR (pmol/min)/DNA(ug/µL)
WT P16t P19bt
l
Glycolysis
ECAR (mpH/min)/DNA (ng/uL)
Ctrl DCA
m
P16t
% γH2AX+ cells
ns
DCA
5-FU
n
APKS - Glucose sensor
10mM glucose
YFP/CFP
time (minutes)
o
Ctrl DCA TEPP-46 TEPP-46 + DCA
DIC
Glucose sensor YFP/CFP
p
YFP/CFP (=glucose)
DCA
TEPP-46

Supplementary Fig. 5. Respiratory phenotype of CRC organoids and metabolic analysis of 2-DG, DCA and TEPP-46 treatments. a and **b).** Basal oxygen consumption rate (OCR) and maximal respiration of WT and CRC organoids determined by mitochondrial stress test by Seahorse XF analysis (mean ± SEM, n = 5, one-way ANOVA). **c)** Determination of extracellular acidification rate (ECAR) during a Seahorse XF glycolysis stress test of APKS organoids treated with 10mM 2-DG for 24 hours (mean ± SEM, 5 technical replicates, representative for n = 3). **d)** Western blot detection of (p)PDH of WT and CRC organoids treated with DCA for 24 hours (blot representative for n = 3). WT, AK, AP, APK and APKS were run together on the same gels. **e)** Extracellular acidification rate (ECAR) of WT, APK and APKS organoids treated with DCA for 24 hours, determined by a glycolysis stress test by Seahorse XF analysis (mean ± SEM, n = 4-7, one-way ANOVA, Sidak's multiple comparisons test). **f** and **g)** Basal and maximal respiration of APK and APKS organoids treated with DCA for 24 hours, determined by a Seahorse XF mitochondrial stress test (OCR is normalized to the non-treated controls, mean ± SEM, n=5-8, one-sample t-test). **h)** Detection of (deoxy)nucleotide triphosphates by metabolomics of APKS organoids treated with DCA for 20 hours (mean ± SEM, n=2 with 3 technical replicates each (from repeated measurements) (dATP only detected in 1 experiment), one-sample t-test). **i)** Quantification of cells with DNA damage by flow cytometry of AK and AP organoids treated with 5-FU for 48 hours. DCA treatment started 20 hours before 5-FU treatment (mean ± SEM, WT: n = 3-6, AP: one-way ANOVA, Sidak's multiple comparisons test, AK: Kruskal-Wallis test, Dunn's multiple comparisons test). **j)** Extracellular acidification rate (ECAR) of WT, P16t and P19bt organoids determined by glycolysis stress test by Seahorse XF analysis (mean ± SEM, n = 4-5, one-way ANOVA, Sidak's multiple comparisons test).

k) Basal oxygen consumption rate (OCR) and maximal respiration of WT, P16t and P19bt organoids determined by mitochondrial stress test by Seahorse XF analysis (mean ± SEM, n =4-6, one-way ANOVA). **l)** Extracellular acidification rate (ECAR) of P19bt organoids treated with 10mM DCA for 24 hours, determined by glycolysis stress test by Seahorse XF analysis (mean ± SEM, n = 4 technical replicates, representative for 2 independent experiments, unpaired t-test). **m)** Quantification of cells with DNA damage by flow cytometry of P16t organoids treated with 5-FU for 48 hours and stained with anti-γH2AX. DCA treatment started 20 hours before 5-FU treatment (mean ± SEM, n = 3, one-way ANOVA, Sidak's multiple comparisons test). **n)** YFP/CFP ratio APKS organoids transduced with a FRET-glucose sensor treated with 10 mM glucose as validation of the glucose sensor (mean ± SEM, 5 technical replicates, YFP/CFP ratio resembles glucose concentration). **o, p)** Representative images of APKS organoids transduced with the FRET-glucose sensor treated with DCA and TEPP-46 for 24 hours (scale bar = 50 µm) (O), and quantification of these images (mean ± SEM, 17-22 organoids from 4 independent experiments, Kruskal-Wallis test, Dunn's multiple comparisons test) (P) (YFP/CFP ratio resembles glucose concentration).

ns: non-significant, * $p < 0.05$, ** $p < 0.01$, *** $p < 0.001$

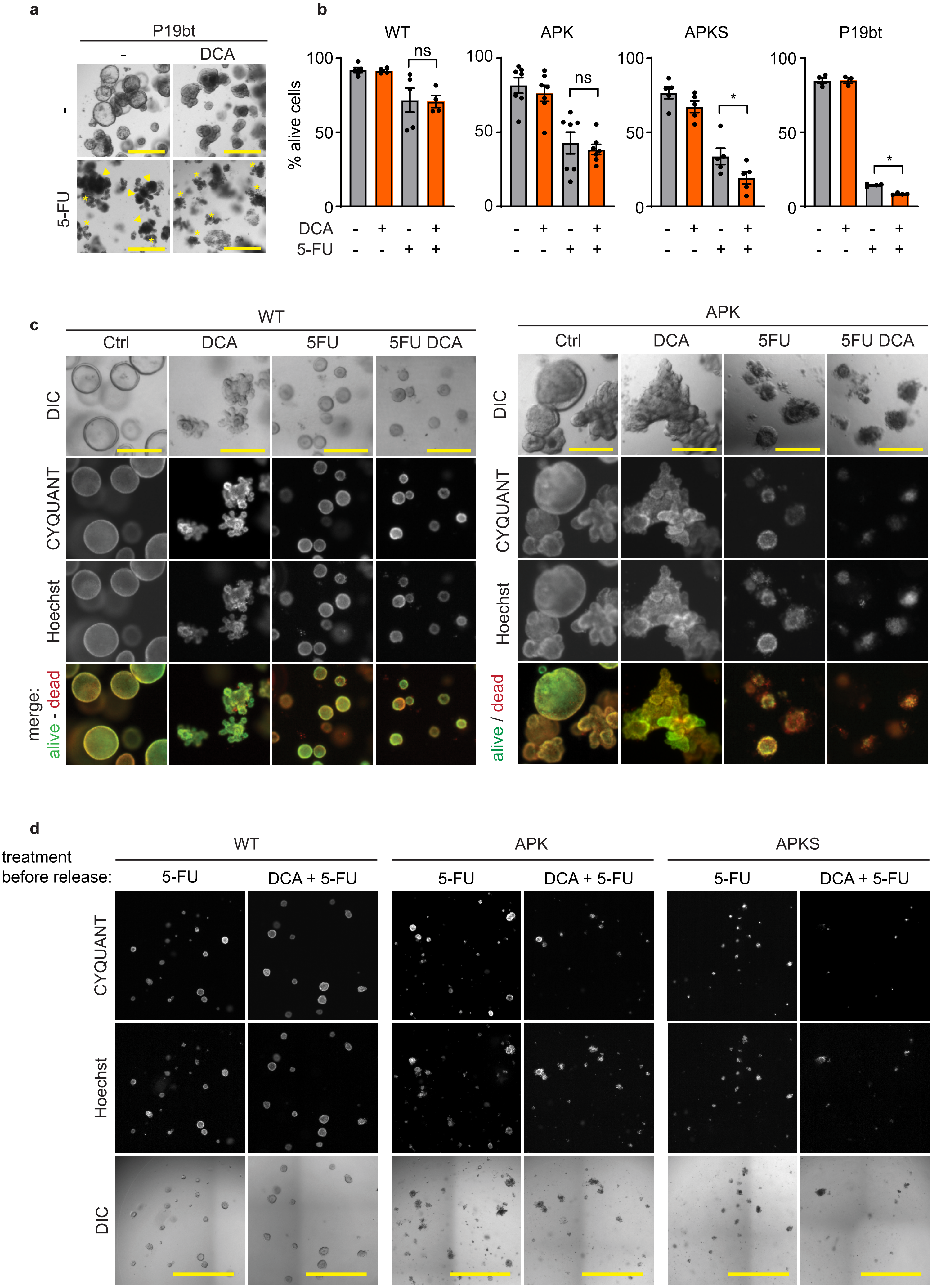
a
P19bt
-
DCA
-
5-FU
b
WT
APK
APKS
P19bt
% alive cells
100
50
0
ns
ns
*
*
DCA
5-FU
c
WT
APK
Ctrl
DCA
5FU
5FU DCA
DIC
CYQUANT
Hoechst
merge: alive - dead
alive / dead
d
treatment before release:
WT
APK
APKS
5-FU
DCA + 5-FU
CYQUANT
Hoechst
DIC

Supplementary Fig. 6. Rewiring glucose metabolism improves 5-FU-induced cytotoxicity specifically in CRC organoids. a) Representative brightfield images of P19bt organoids treated with 5-FU for 7 days. DCA (10 mM) treatment was started 20 hours before 5-FU administration (scale bar = 500 µm, arrow heads indicate survivor organoids, asterisks indicate compromised organoids). **b)** Cell viability analysis of WT and CRC tumor organoids by flow cytometry to distinguish alive ($DAPI^-$) from death cells ($DAPI^+$) upon 7 days of 5-FU treatment. DCA treatment (20 mM for WT, APK and APKS and 10 mM for P19bt) started 20 hours before 5-FU administration (mean ± SEM, n = 4-7, one-way ANOVA, Sidak's multiple comparisons test). **c)** Representative images of WT and APK organoids, stained with CYQUANT (alive) and Hoechst (total), treated with 5-FU for 7 days. DCA treatment started 20 hours before 5-FU treatment (scale bar = 300 µm). **d)** Representative images of WT, APK and APKS organoids 4 days after replating, upon a 48 hour-5-FU (50 µM) treatment (with or without DCA treatment that started 20 hours before 5-FU administration), followed by 7 days of recovery time. Organoids were stained with CYQUANT (alive) and hoechst (total)(scale bar = 1 mm).

ns: non significant, * $p < 0.05$

Supplementary Fig. 7. Uncropped and unedited blot images corresponding to the western blots presented in all figures and supplmentary figures of the mansucript.

Figure 1c

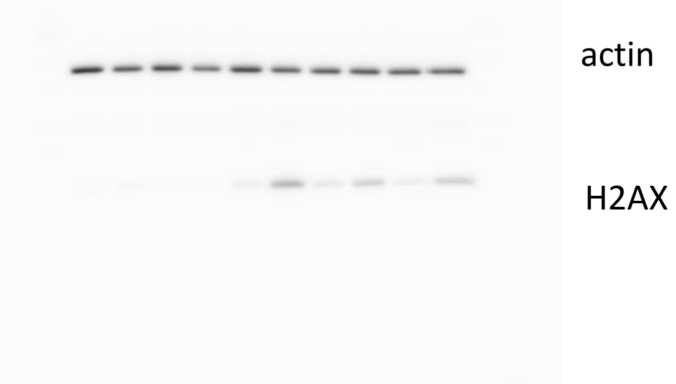

Figure 1e

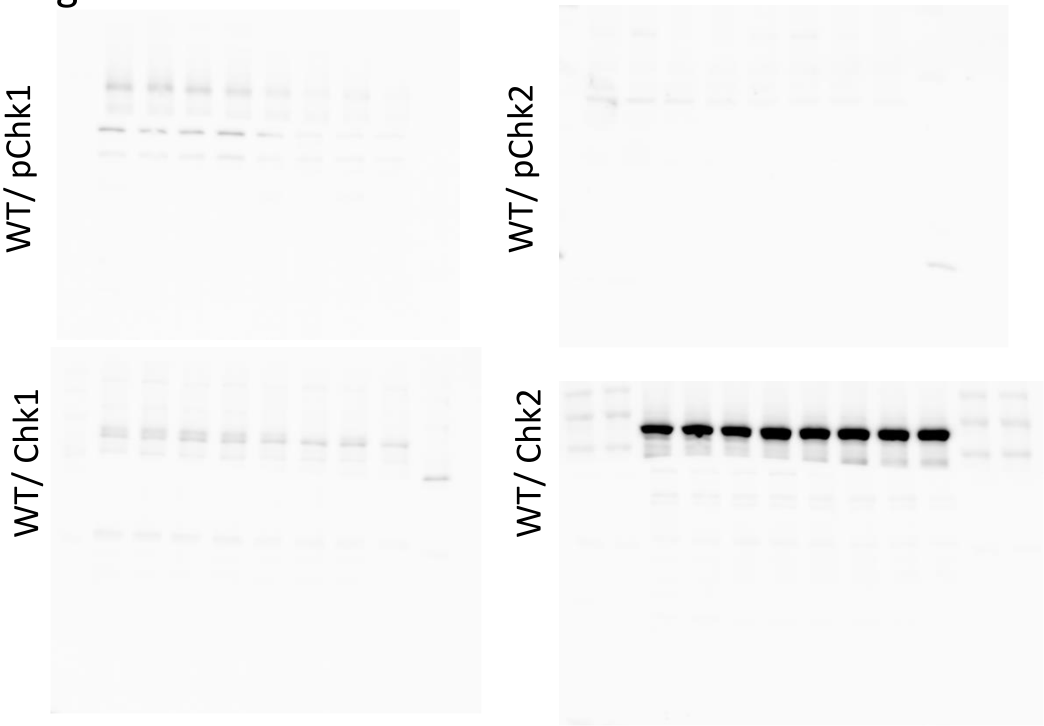

Figure 1e

APKS/ pChk1

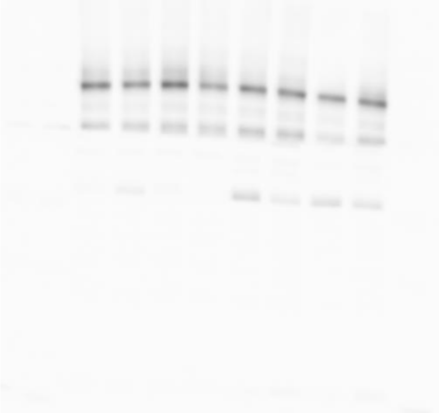

APKS/ pChk2

APKS/ Chk1

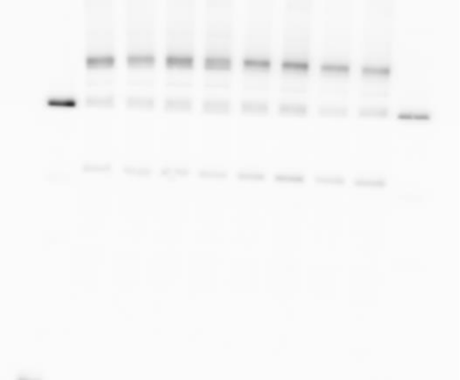

APKS/ Chk2

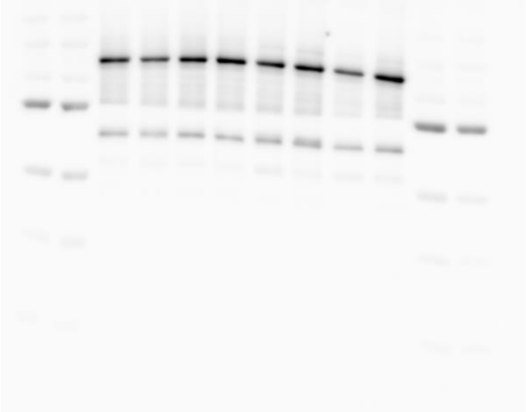

Figure 1f

WT/ H2Ax

APKS/ H2Ax

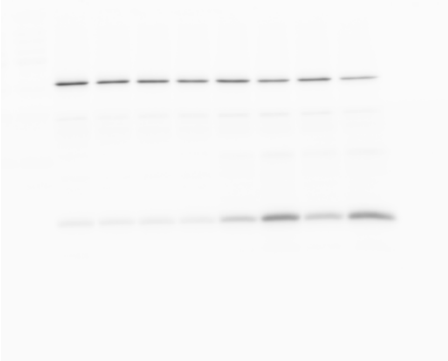

WT/ vinculin

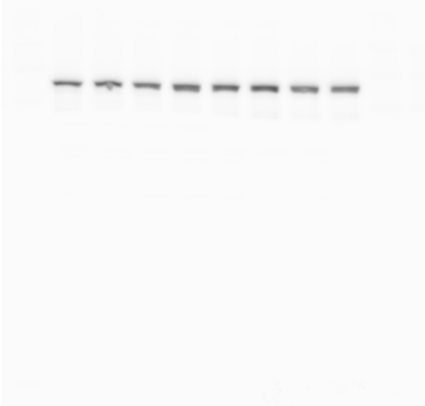

APKS/ vinculin

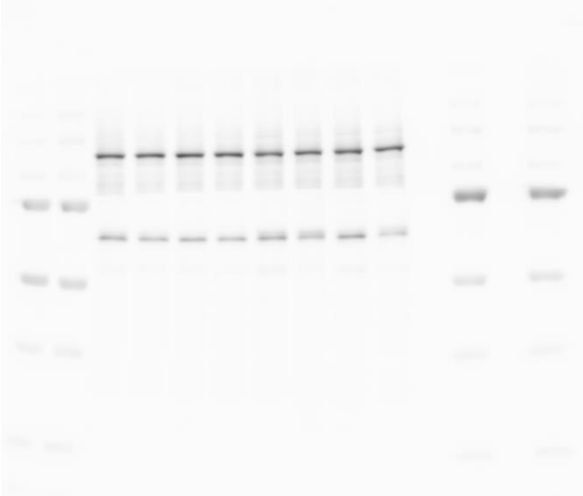

Figure 3a

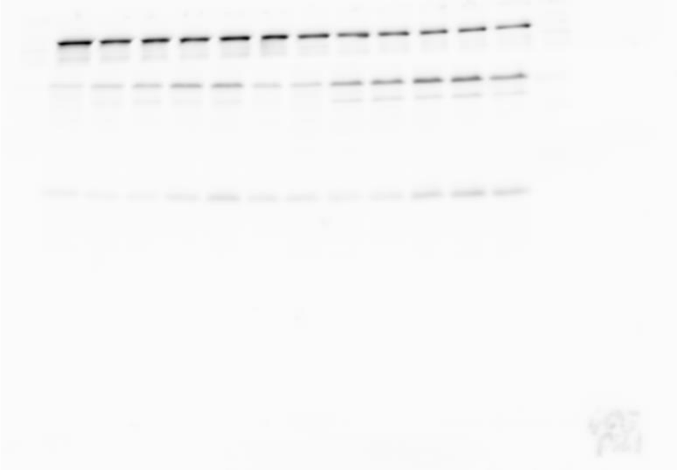

WT and AK/ Vinculin

WT and AK/ p53

WT and AK/ p21

Figure 3b

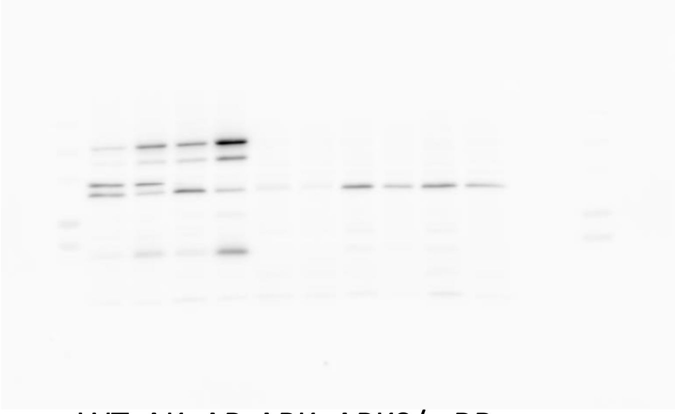

WT, AK, AP, APK, APKS/ p53

WT, AK, AP, APK, APKS/ p21

WT, AK, AP, APK, APKS/ pRB

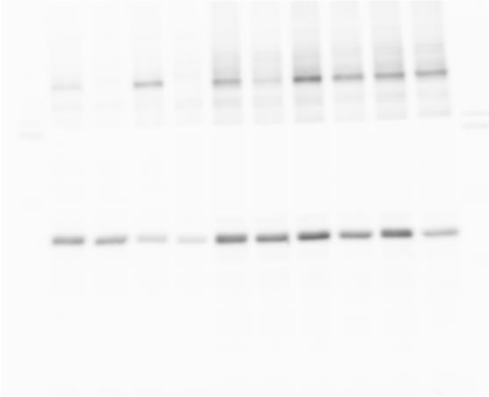

WT, AK, AP, APK, APKS/ RB

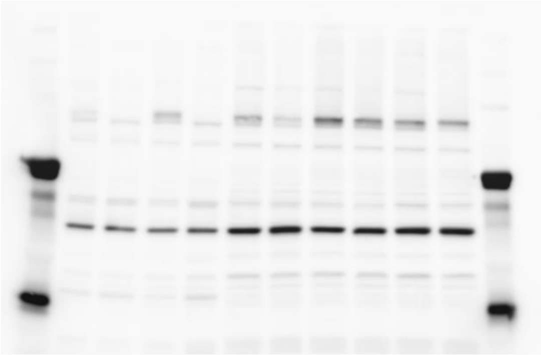

WT, AK, AP, APK, APKS/ Vinculin upper panel

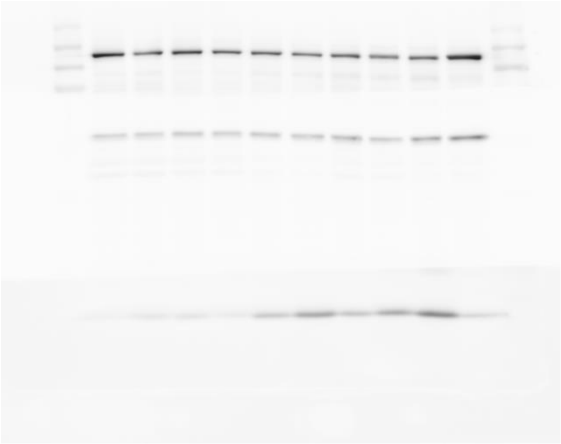

WT, AK, AP, APK, APKS/ Vinculin lower panel

Figure 3d

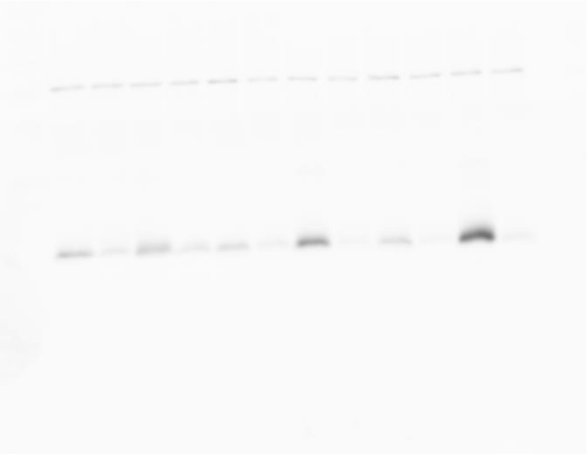

AP, APK, APKS/ H2AX

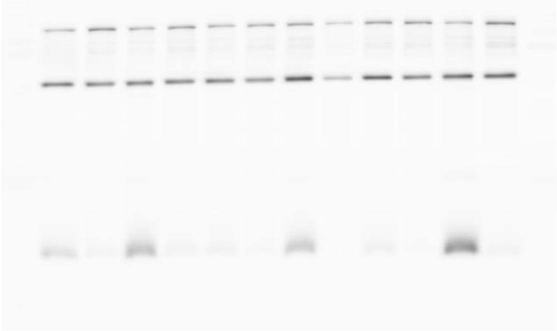

AP, APK, APKS/ Tubulin

Figure 3g (APK and APKS P53 OE)

lanes 1-4

APK/ p53

APK/ p21

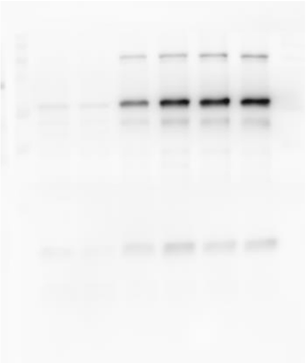

lanes 1-4

APK/ H2AX

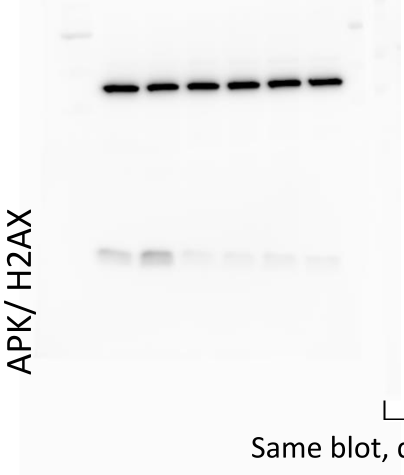

lanes 1-4

APK/ actin

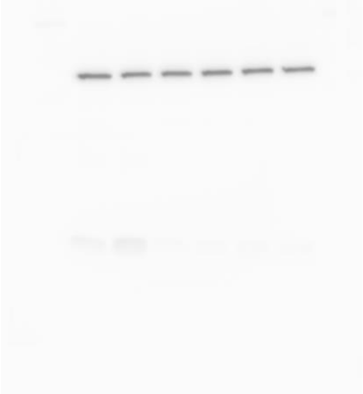

Same blot, different exposure time

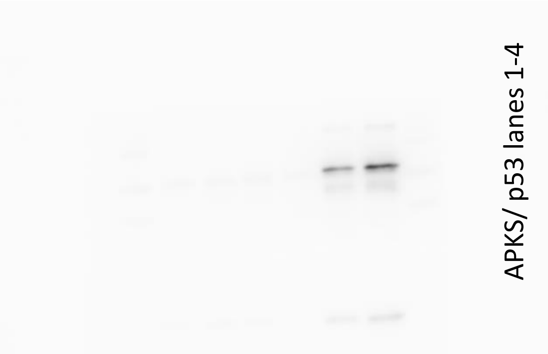

APKS/ p21

APKS/ p53 lanes 1-4

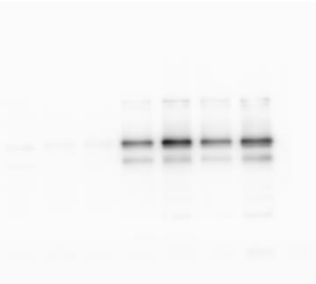

APKS/ actin lanes 3-6

APKS/ H2AX lanes 3-6

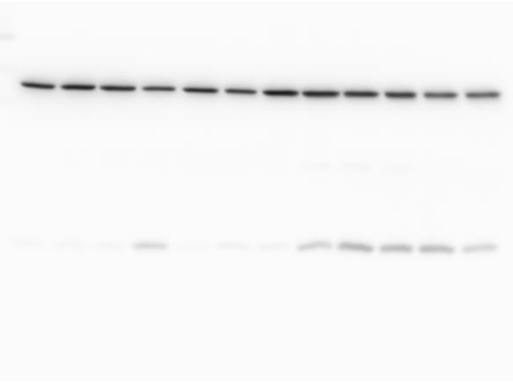

Figure 4a

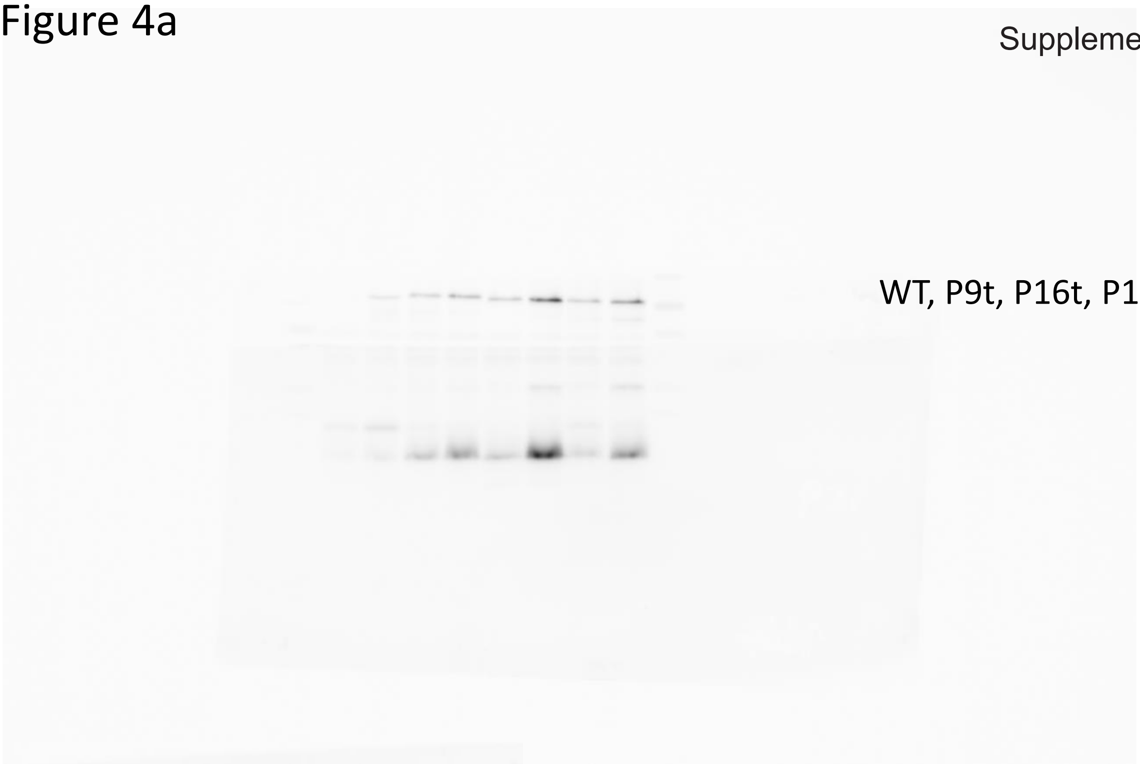

WT, P9t, P16t, P19bt/ p53

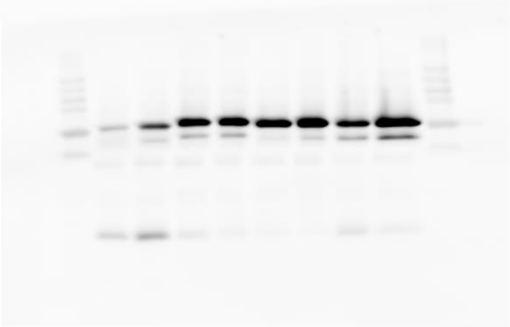

WT, P9t, P16t, P19bt/ p21

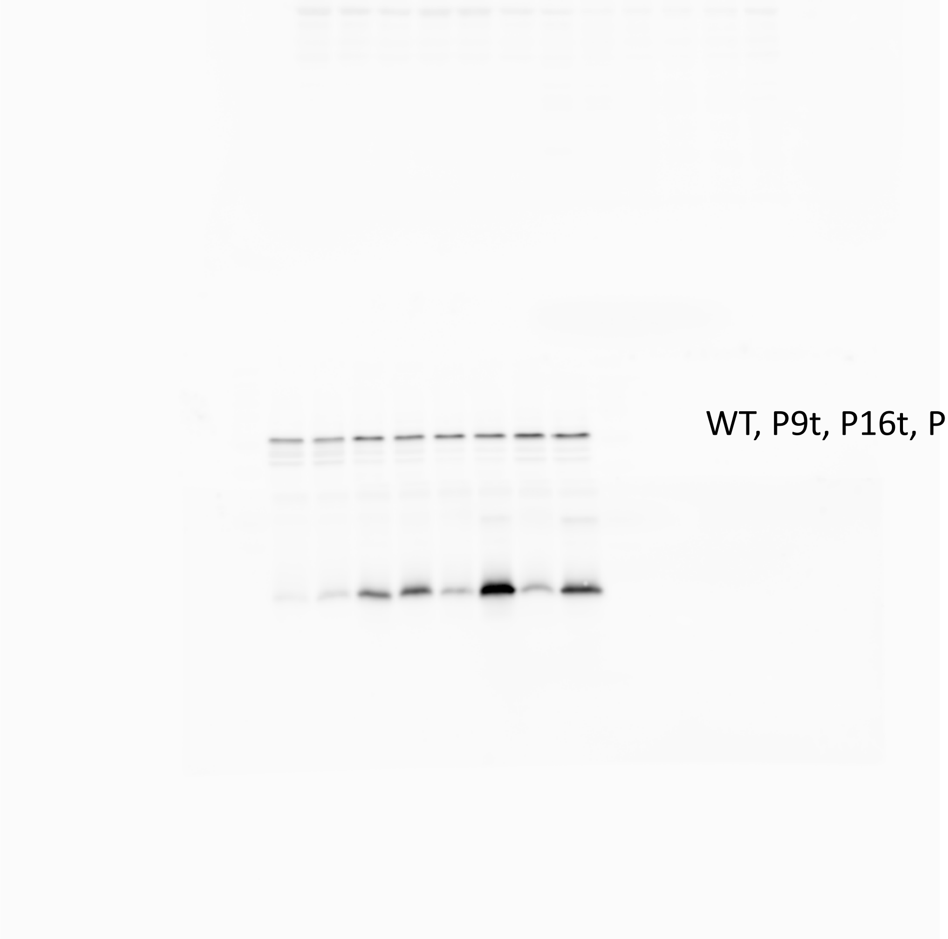

WT, P9t, P16t, P19bt/ Tubulin

Figure 4f

P16t-P53OE, P19bt-P53OE/ p53

P16t-P53OE, P19bt-P53OE/ p21

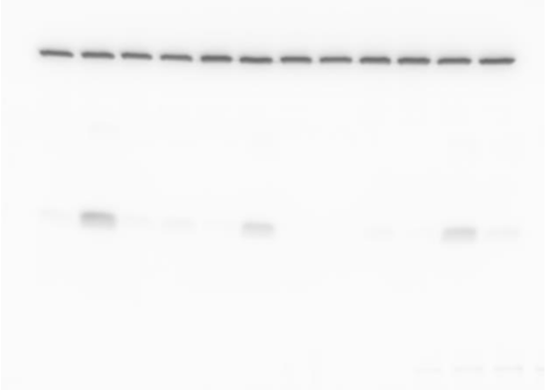

P16t-P53OE, P19bt-P53OE/ actin

P16t-P53OE, P19bt-P53OE/ H2AX

Figure 5b

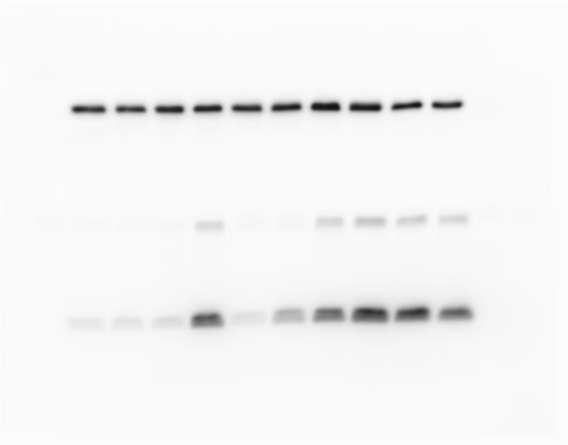

Tubulin lanes 1-6

H2AX lanes 1-6

Suppl. Fig 1e

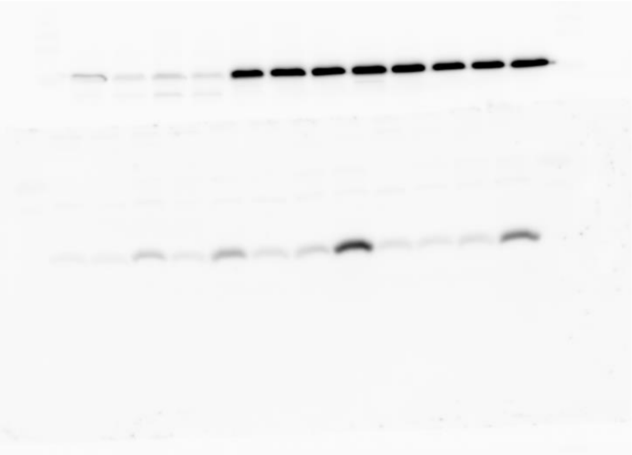

WT, AP, APK/ H2AX

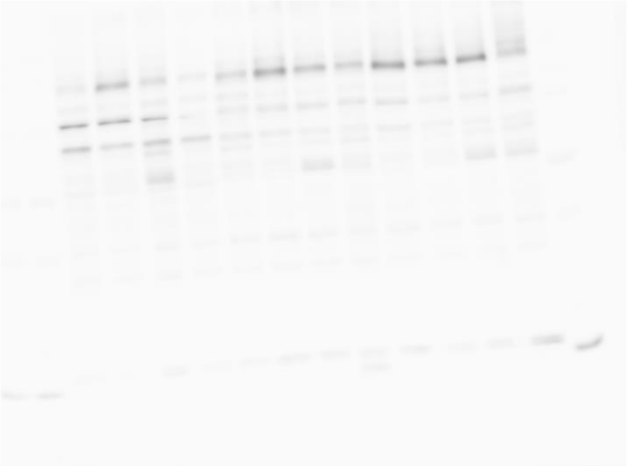

WT, AP, APK/ pChk1

WT, AP, APK/ pChk2

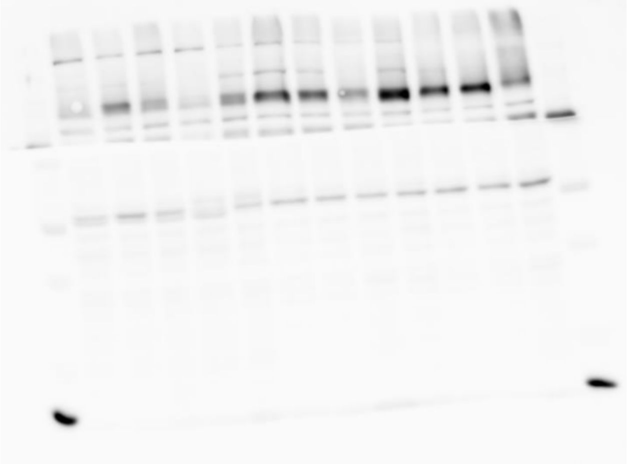

WT, AP, APK/ Chk1

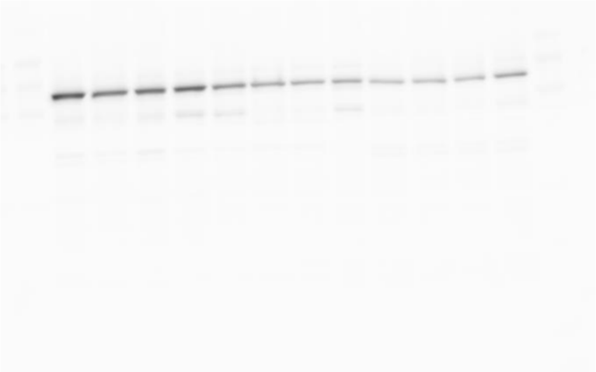

WT, AP, APK/ Chk2

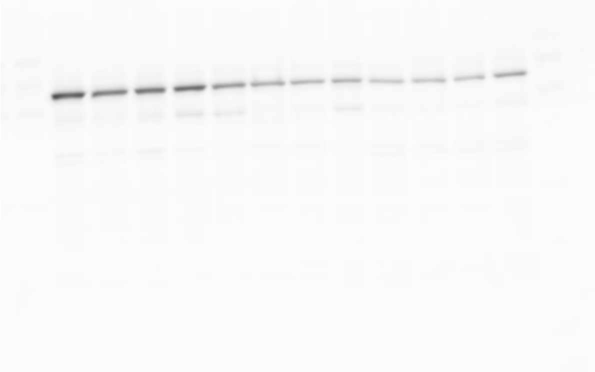

WT, AP, APK/ Vinculin

Suppl. Fig 1e

AK/ pChk1 lanes 5-8

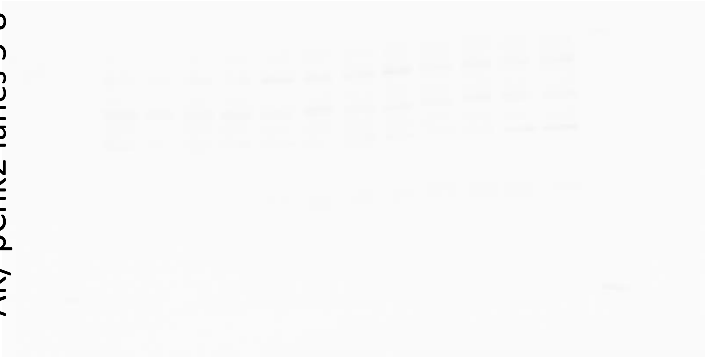

AK/ pChk2 lanes 5-8

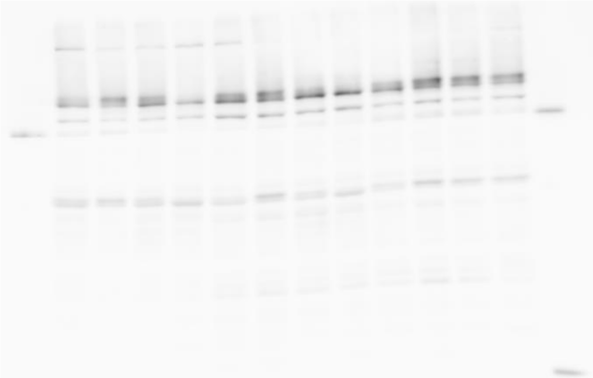

AK/ Chk1 lanes 5-8

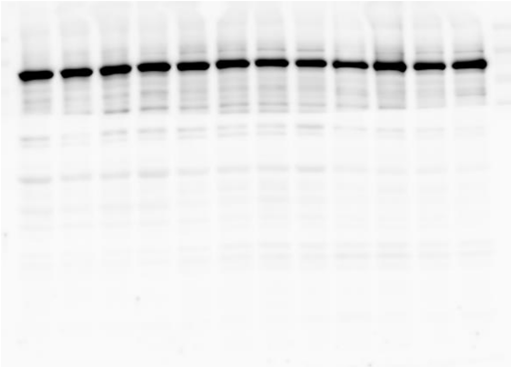

AK/ Chk2 lanes 5-8

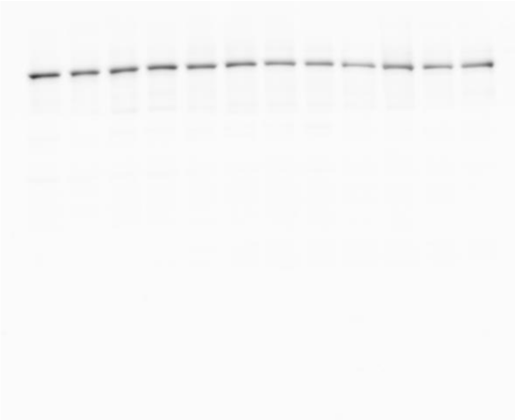

AK/ Vinculin lanes 5-8

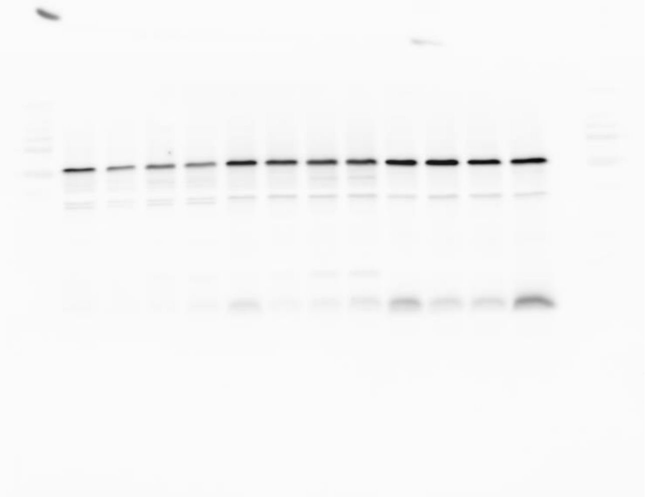

AK/ H2AX lanes 5-8

Suppl. Fig 1e

APKS/ pChk1

APKS/ pChk2

APKS/ Chk1

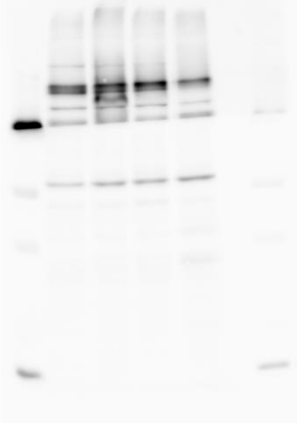

APKS/ Chk2

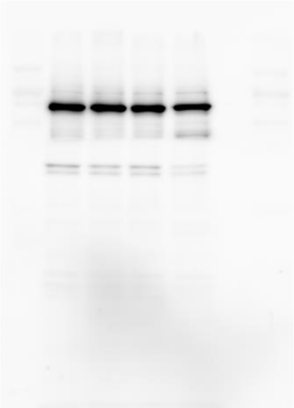

APKS/ Vinculin

APKS/ H2AX lanes 5-8

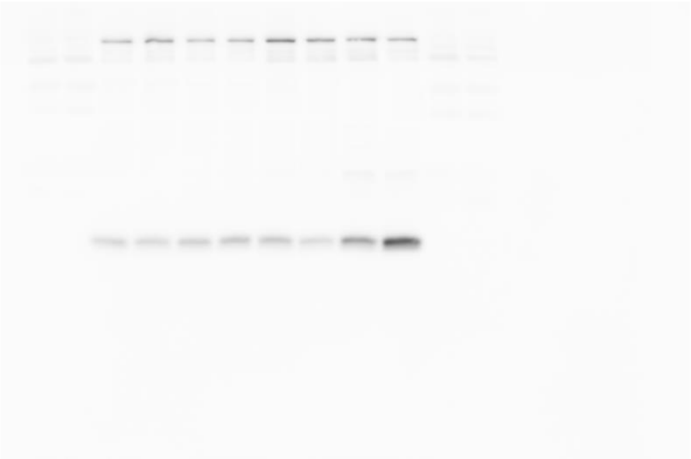

Suppl. Fig 4b

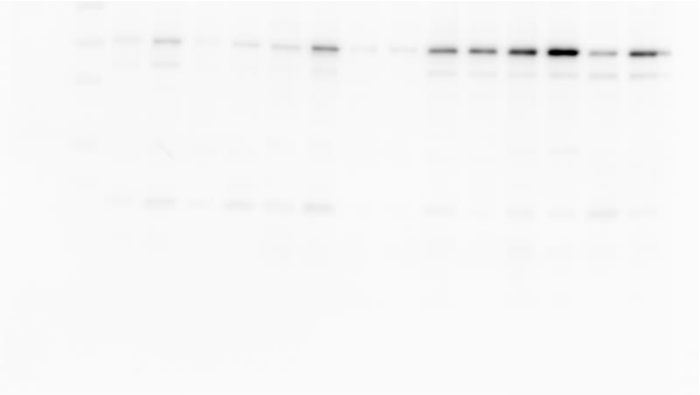

WT, P7t, P14t/ p53 lanes 1-6

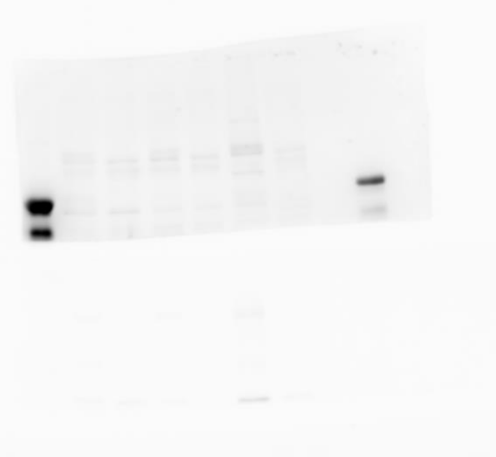

WT, P7t, P14t/ Rb

WT, P7t, P14t/ pRb

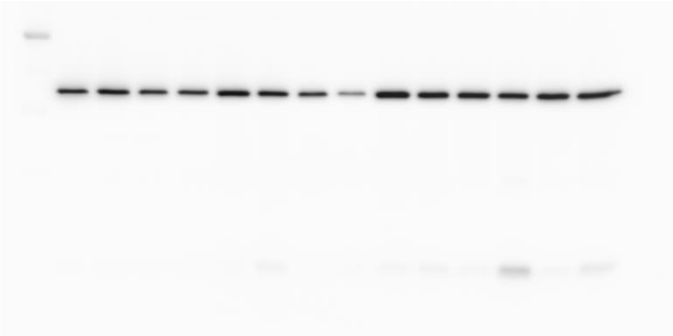

WT, P7t, P14t/ actin lanes 1-6

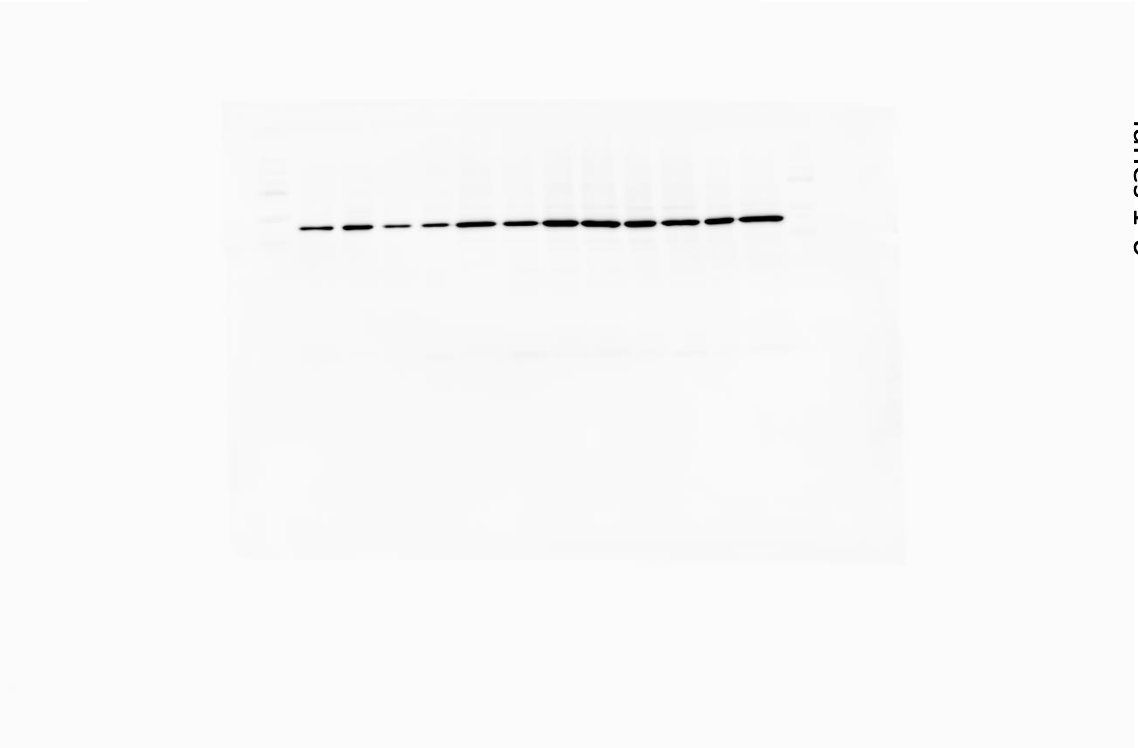

WT, P7t, P14t/ cleaved caspase3 lanes 1-6

Suppl. Fig 5d

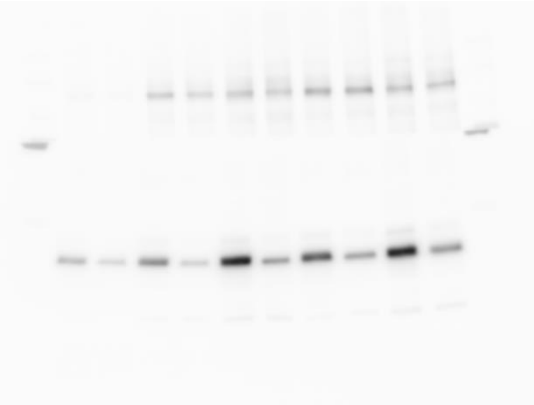

WT, AK, AP, APK, APLS/ pPDH

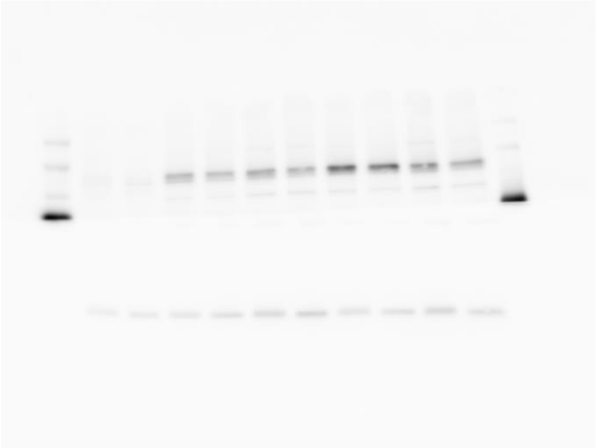

WT, AK, AP, APK, APLS/ PDH
