# Supplementary material for: Rewiring glucose metabolism improves 5-FU efficacy in p53-deficient/KRASG12D glycolytic colorectal tumors
Source: Commun Biol. 2022 Oct 31;5:1159. doi: 10.1038/s42003-022-04055-8 (PMC9622833; doi:10.1038/s42003-022-04055-8)
Supplement: Supplementary file 3 — Description of Additional Supplementary Files [file 42003_2022_4055_MOESM3_ESM.pdf]

Description of Additional Supplementary Files

File name: Supplementary Data 1
Description: The source data behind the graphs in the main figures of the paper.

File name: Supplementary Data 2
Description: The source data behind the graphs in the supplementary figures of the paper.
