# Supplementary material for: Rewiring glucose metabolism improves 5-FU efficacy in p53-deficient/KRASG12D glycolytic colorectal tumors
Source: Commun Biol. 2022 Oct 31;5:1159. doi: 10.1038/s42003-022-04055-8 (PMC9622833; doi:10.1038/s42003-022-04055-8)
Supplement: Supplementary file 6 — Reporting Summary [file 42003_2022_4055_MOESM6_ESM.pdf]

nature research

Corresponding author(s): M.J. Rodriguez Colman

Last updated by author(s): 04-05-2022

Reporting Summary

Nature Research wishes to improve the reproducibility of the work that we publish. This form provides structure for consistency and transparency in reporting. For further information on Nature Research policies, see our Editorial Policies and the Editorial Policy Checklist.

Statistics

For all statistical analyses, confirm that the following items are present in the figure legend, table legend, main text, or Methods section.

n/a	Confirmed	
☐	☒	The exact sample size (*n*) for each experimental group/condition, given as a discrete number and unit of measurement
☐	☒	A statement on whether measurements were taken from distinct samples or whether the same sample was measured repeatedly
☐	☒	The statistical test(s) used AND whether they are one- or two-sided<br>*Only common tests should be described solely by name; describe more complex techniques in the Methods section.*
☒	☐	A description of all covariates tested
☐	☒	A description of any assumptions or corrections, such as tests of normality and adjustment for multiple comparisons
☒	☐	A full description of the statistical parameters including central tendency (e.g. means) or other basic estimates (e.g. regression coefficient) AND variation (e.g. standard deviation) or associated estimates of uncertainty (e.g. confidence intervals)
☒	☐	For null hypothesis testing, the test statistic (e.g. *F*, *t*, *r*) with confidence intervals, effect sizes, degrees of freedom and *P* value noted<br>*Give P values as exact values whenever suitable.*
☒	☐	For Bayesian analysis, information on the choice of priors and Markov chain Monte Carlo settings
☒	☐	For hierarchical and complex designs, identification of the appropriate level for tests and full reporting of outcomes
☒	☐	Estimates of effect sizes (e.g. Cohen's *d*, Pearson's *r*), indicating how they were calculated

Our web collection on statistics for biologists contains articles on many of the points above.

Software and code

Policy information about availability of computer code

Data collection	No software was used
Data analysis	No software was used

For manuscripts utilizing custom algorithms or software that are central to the research but not yet described in published literature, software must be made available to editors and reviewers. We strongly encourage code deposition in a community repository (e.g. GitHub). See the Nature Research guidelines for submitting code & software for further information.

Data

Policy information about availability of data

All manuscripts must include a data availability statement. This statement should provide the following information, where applicable:

- Accession codes, unique identifiers, or web links for publicly available datasets
- A list of figures that have associated raw data
- A description of any restrictions on data availability

All data generated or analysed during this study are included in this published article (and its supplementary information files).

Field-specific reporting

Please select the one below that is the best fit for your research. If you are not sure, read the appropriate sections before making your selection.

☒ Life sciences ☐ Behavioural & social sciences ☐ Ecological, evolutionary & environmental sciences

For a reference copy of the document with all sections, see nature.com/documents/nr-reporting-summary-flat.pdf

Life sciences study design

All studies must disclose on these points even when the disclosure is negative.

Sample size	Due to the nature of this study, no elaborated sample-size calculation was performed. Selected sample size (referring to the number of independent experiments) allowed application of statistical analysis (parametric or non-parametric testing upfront).
Data exclusions	No data was excluded, with the exception of technically failures.
Replication	Independent repetition of experiments was performed at least three times to ensure reproducibility. Repetition of experiments were performed by the first and second author together or independently. The attempts at replication were successful.
Randomization	Allocation of the treatments were randomly distributed into experimental groups.
Blinding	Investigators were in general not blinded during data collection and analysis, with the exception of the data collection and analysis percformed by research technicians. Western blotting, flow cytometry and Seahorse XF analysis analytic methods are standardized and minimize bias. Quantification of immunofluorescent and CYQUANT stainings is an automatized method that did not require blinding.

Reporting for specific materials, systems and methods

We require information from authors about some types of materials, experimental systems and methods used in many studies. Here, indicate whether each material, system or method listed is relevant to your study. If you are not sure if a list item applies to your research, read the appropriate section before selecting a response.

Materials & experimental systems

n/a	Involved in the study	
☐	☒	Antibodies
☐	☒	Eukaryotic cell lines
☒	☐	Palaeontology and archaeology
☒	☐	Animals and other organisms
☒	☐	Human research participants
☒	☐	Clinical data
☒	☐	Dual use research of concern

Methods

n/a	Involved in the study	
☒	☐	ChIP-seq
☐	☒	Flow cytometry
☒	☐	MRI-based neuroimaging

Antibodies

Antibodies used	vinculin (Sigma, #V9131), Tubulin (Merck Millipore, #CP06 OS), γH2AX (Sigma, #05-636), pChk1(S345) (Cell Signaling, #2348, Chk1 (Santa Cruz, #SC-8408), pChk2(T68) (Cell Signaling, #2661), Chk2 (Cell Signaling, #3440 and Santa Cruz, #SC-9064), pRb(S780) (Cell Signaling, #9307), Rb (Santa Cruz, #SC-7905), p53 (Santa Cruz, #SC-126), p21 (BD Bioscience, #556430), pPDH(S293) (Abcam, #ab92696), PDH (Invitrogen, #459400).
Validation	Antibodies were validated by the manufacturers and relevant information can be found in their websites.

Eukaryotic cell lines

Policy information about cell lines

Cell line source(s)	Organoid lines. Tumor progression organoids were a gift from the Hans Clervers Lab. Patient derived organoids P7t, P9t, P14t, P16t and, P19bt were obtained from the HUB biobank.
Authentication	Performed 2-3 times during the duration of the study based on sequencing of specific regions (TPOs) and by SNPs sequencing (PDOs).
Mycoplasma contamination	Performed routinely every 4-6 months.

Commonly misidentified lines (See ICLAC register)	N/A

Flow Cytometry

Plots

Confirm that:

☒ The axis labels state the marker and fluorochrome used (e.g. CD4-FITC).

☒ The axis scales are clearly visible. Include numbers along axes only for bottom left plot of group (a 'group' is an analysis of identical markers).

☒ All plots are contour plots with outliers or pseudocolor plots.

☒ A numerical value for number of cells or percentage (with statistics) is provided.

Methodology

Sample preparation	Organoids were collected in ice-cold DMEM/F12 medium, supplemented with penicillin/streptomycin, 10 mM HEPES and 1x Glutamax (DMEM/F12 +++ medium) and subsequently incubated with trypsin (Sigma). For cell viability analysis, cells were stained with DAPI (Sigma-Aldrich, #D9564) on ice for 5 minutes and immediately analyzed by flow cytometry. Viability was determined by DAPI staining, where DAPI- cells were considered alive, and DAPI+ cell s as dead. For γH2AX staining, cell cycle profile analysis and EdU incorporation analysis, single cells were fixed in (PFA) (#1004965000, Merck Millipore) at RT for 10 min and permeabilized overnight on ice with 70% EtOH. For γH2AX staining, cell cycle profile analysis, organoids were washed once with 10 mL PBS + 1% BSA and 0.02% tween, incubated with Phospho-Histone H2A.X (Ser 139)-Alexa Fluor 488 (eBioscience, #53-9865-82) for 30 min at RT, covered from light. For cell cycle profiling, organoids were incubated with RNAse (100 µg/ml) in PBS for 20 minutes at RT and with DAPI for 2 hours, on ice, covered from light. For EdU detection, cells were stained by the Click-iT Plus EdU Pacific Blue Flow cytometry assay kit (Thermo fisher, #C10636) according to manufacturer's instructions.
Instrument	BD FACS Celesta #660345
Software	BD FACSDiva software
Cell population abundance	N.A.
Gating strategy	The gating strategy can be found in supplementary figure 1 and 2

☒ Tick this box to confirm that a figure exemplifying the gating strategy is provided in the Supplementary Information.
